# Melatonin Inhibits NF-κB/CREB/Runx2 Signaling and Alleviates Aortic Valve Calcification

**DOI:** 10.3389/fcvm.2022.885293

**Published:** 2022-06-20

**Authors:** Shao-Jung Li, Wan-Li Cheng, Yu-Hsun Kao, Cheng-Chih Chung, Nguyen Ngoc Trang, Yi-Jen Chen

**Affiliations:** ^1^Division of Cardiovascular Surgery, Department of Surgery, School of Medicine, College of Medicine, Taipei Medical University, Taipei, Taiwan; ^2^Division of Cardiovascular Surgery, Department of Surgery, Wan Fang Hospital, Taipei Medical University, Taipei, Taiwan; ^3^Cardiovascular Research Center, Wan Fang Hospital, Taipei Medical University, Taipei, Taiwan; ^4^Taipei Heart Institute, Taipei Medical University, Taipei, Taiwan; ^5^Graduate Institute of Clinical Medicine, College of Medicine, Taipei Medical University, Taipei, Taiwan; ^6^Department of Medical Education and Research, Wan Fang Hospital, Taipei Medical University, Taipei, Taiwan; ^7^Division of Cardiovascular Medicine, Department of Internal Medicine, Wan Fang Hospital, Taipei Medical University, Taipei, Taiwan; ^8^Division of Cardiology, Department of Internal Medicine, School of Medicine, College of Medicine, Taipei Medical University, Taipei, Taiwan; ^9^Radiology Center, Bach Mai Hospital, Hanoi, Vietnam

**Keywords:** melatonin, osteogenesis, NF-κB, calcific aortic valve disease, cyclic AMP response element-binding protein, runt-related transcription factor 2, valvular interstitial cell

## Abstract

Calcific aortic valve disease (CAVD) is linked to high mortality. Melatonin inhibits nuclear factor-kappa B (NF-κB)/cyclic AMP response element-binding protein (CREB), contributing to CAVD progression. This study determined the role of melatonin/MT1/MT2 signaling in valvular interstitial cell (VIC) calcification. Western blotting and Alizarin red staining were used to analyze NF-κB/CREB/runt-related transcription factor 2 (Runx2) signaling in porcine VICs treated with an osteogenic (OST) medium without (control) or with melatonin for 5 days. Chromatin immunoprecipitation (ChIP) assay was used to analyze NF-κB's transcription regulation of NF-κB on the Runx2 promoter. OST medium-treated VICs exhibited a greater expression of NF-κB, CREB, and Runx2 than control VICs. Melatonin treatment downregulated the effects of the OST medium and reduced VIC calcification. The MT1/MT2 antagonist (Luzindole) and MT1 receptor neutralized antibody blocked the anticalcification effect of melatonin, but an MT2-specific inhibitor (4-P-PDOT) did not. Besides, the NF-κB inhibitor (SC75741) reduced OST medium-induced VIC calcification to a similar extent to melatonin at 10 nmol/L. The ChIP assay demonstrated that melatonin attenuated OST media increased NF-κB binding activity to the promoter region of Runx2. Activation of the melatonin/MT1-axis significantly reduced VIC calcification by targeting the NF-κB/CREB/Runx2 pathway. Targeting melatonin/MT1 signaling may be a potential therapeutic strategy for CAVD.

## Introduction

Calcific aortic valvular disease (CAVD) is the most common valvular heart disease worldwide. Although aging is dominant in CAVD development, its molecular mechanisms require further elucidation. Pathogenesis is complicated and multifactorial and can include inflammation, extracellular matrix (ECM) change, fibrosis, and calcification. Osteogenesis of aortic valvular interstitial cells (VICs) constitutes a significant risk factor in CAVD (1). Myofibroblast-like VICs are also involved in ECM remodeling (2). Runt-related transcription factor 2 (Runx2) plays a role in osteoblast differentiation during embryonic development and acts as a critical transcription regulator of bone matrix protein deposition in postnatal life, inducing osteogenesis-associated gene expression in osteogenic tissue (3, 4). Elevation of nuclear factor-kappa B (NF-κB) signaling is a critical mediator of inflammation involved in CAVD pathogenesis (5). NF-κB is inactive in the cytoplasm due to the inhibitory effect of the IκBs (6). The canonical NF-κB pathway is activated by numerous inflammatory stimuli to induce phosphorylation of the IκB proteins, thereby inducing IκB ubiquitination and degradation activities. NF-κB is activated in the aortas of diabetic mice and serves as the transcription factor of Runx2 (7).

Melatonin (N-acetyl-5-methoxytryptamine) and its metabolites could inhibit the NF-κB pathway. Melatonin is an anti-inflammatory agent in various neurological diseases, including multiple sclerosis, Parkinson's disease, brain ischemia, stroke, and reperfusion. As an indoleamine formed in the pineal gland and secreted into the blood predominantly during the night, melatonin is also a plant-derived product that exhibits antioxidant, anti-tumor, and immunomodulation effects. Studies have reported that melatonin can positively affect bone metabolism, though its osteogenic mechanism remains unclear. Melatonin has various receptors, including G protein-coupled classical melatonin (melatonin receptor 1/melatonin receptor 2, MT1/MT2) and nuclear receptors (ROR/RZR) (8, 9). Melatonin/MT2 increases bone marrow mesenchymal stem cell osteogenic action but inhibits their mediated osteoclastogenesis through inhibiting NF-κB signaling (10). Melatonin can also reduce the phosphorylation of cyclic AMP response element-binding protein (CREB) (11, 12). CREB phosphorylation and the subsequent transactivation of the cAMP response element can result in c-fos expression, which binds to the promoter region of Runx2 and induces osteoblast differentiation (13). In this study, we analyzed the effects of melatonin on VIC calcification and explored the underlying mechanisms.

## Materials and Methods

### Isolation and Culture of Primary Porcine VICs

The animal study was approved by Taipei Medical University's Institutional Animal Care and Use Committee (LAC-2020-0332). The slaughterhouse (Yahsen Frozen Foods, Taoyuan, Taiwan) followed the Humane Slaughter Act guidelines and the Animal Protection Act for the care and slaughter of swine. We obtained the aortic valve leaflets of a 6-month-old pig from a slaughterhouse. The porcine VICs were isolated using collagenase I (250 U/mL; Sigma-Aldrich, St. Louis, MO, USA) and treated as the previous study revealed (14). The culture media and chemicals were changed daily, and the VICs were seeded at a density of 2 × 10^4^ cells/cm^2^ in six-well plates. The aortic VICs from passage three-five were used for further experiments.

### Osteogenesis of Aortic VICs

Aortic VICs that reached 80% confluence were cultured in an osteogenic (OST) medium with a DMEM/F12 complete medium containing 50-mg/mL ascorbic acid, 10-mmol/L β-glycerophosphate, and 10-nmol/L dexamethasone for 5 days with or without melatonin (0.1–10 nmol/L), SC75741 (NF-κB inhibitor, 1 μmol/L), Luzindole (MT1/MT2 receptor inhibitor, 1 μmol/L), 4-P-PDOT (MT2 melatonin receptor antagonist, 1 μmol/L), and anti-MT1 antibody (0.5, 1, 2 μg/mL; Mel-1A-R; 1:5000; Cat# sc-390328, Santa Cruz) or nonspecific immunoglobulin (control IgG, 1 h prior to OST or melatonin treatment) as previously described (15). We confirmed that the expression of MT1 in the porcine VIC was shown in Supplementary Figure 1.

### Alizarin Red S Staining

Alizarin red S (ARS) staining was used to measure calcium deposition. The VICs were fixed with 4% paraformaldehyde for 20 min and then incubated in 2% ARS solution (pH 4.2) for 1 hour. The excessive dye was washed and removed with distilled water. The stained cells were observed using an Olympus CKX41 inverted phase-contrast microscope (Tokyo, Japan). For semiquantitative analysis, we randomly took three images of stained cells from each treatment, and the average staining intensity was analyzed using ImageJ (1.53c version, National Institutes of Health; http://rsb.info.nih.gov/ij/) at 40× magnification (14).

### Western Blotting

VICs were lysed in a protein extraction reagent (Thermo Scientific, Waltham, MA, USA). An equal amount of total protein from each treatment of the VICs was electrophoresed on a sodium dodecyl sulfate-polyacrylamide gel electrophoresis gradient gel (5%−12%) and transferred to an equilibrated polyvinylidene difluoride membrane (Amersham Biosciences, Little Chalfont, UK). The blots were then probed with primary anti-Runx2 antibody (1:1000; Cat# ab23981, Abcam, Cambridge, UK), anti-phosphorylated cyclic AMP response element-binding protein (pCREB)/CREB (pCREB, 1:1500; Cat# 06–519, Millipore, RRID:AB_310153; CREB, 1:2000, Cat# 9197s, Cell Signaling, RRID:AB_331277), and anti-phosphorylated nuclear factor-kappa B (pNF-κB)/NF-κB (1:5000; Cat# 3033/# 8242, Cell Signaling, RRID:AB_331284/RRID:AB_10859369), anti-MT1 antibody (Mel-1A-R; 1:5000; Cat# sc-390328, Santa Cruz), and β-actin (1:20,000; Cat# ab6276, Abcam, RRID:AB_2223210) was used as the internal control. The number of Western immunoblot observations is described in the Figure legend.

### Chromatin Immunoprecipitation Assays

Following the manufacturer's instructions, the chromatin immunoprecipitation (ChIP) analysis was conducted using an EZ-ChIP assay kit (Cat# 17–295, Millipore). First, cell pellets were lysed in lysis buffer with protease inhibitors for 30 min, and genomic DNA fragmentation was conducted using a sonicator. We retained 1% of the total DNA as the input control DNA. Then, the lasting cell lysates were precleared through the addition of salmon sperm DNA/protein-A agarose slurry for 1 h, followed by immunoprecipitation using 5 μg of antibodies against NF-κB (p65) antibodies (Cat# 8242, Cell Signaling, RRID:AB_10859369) or non-specific immunoglobulin (IgG; R & D systems) as a negative control for 18 h at 4 °C with rotation. Next, samples were mixed with protein-A agarose, washed, and eluted bound DNA. Sonication was performed under 3 watts, 30 sec ON, and 30 sec OFF for various cycles (three cycles). The cycling conditions were set as follows: an initial denaturation step of 95 °C for 2 min, followed by 40 cycles of denaturation at 95 °C for 30 sec, 60 °C for 30sec, and 68 °C for 60sec, with additional 2 min at 68 °C. The immunoprecipitated DNA fragments were amplified through PCR using the porcine Runx2 promoter-specific primers: Pig_Runx2 promoter (−127)_F2: 5′-CCTTCTGGATGCCAGGAAGG-3′; and Pig_Runx2 promoter (−10)_R2: 5′-CCTACCACTGTGGCTTTCCC-3′, which were predicted to contain the NF-κB binding sites (−29/−19) (16).

### Statistical Analyses

All results are presented as mean ± standard errors of the mean. Comparisons among VICs at different settings were analyzed using a one-way repeated analysis of variance with posthoc Tukey's test with the Shapiro-Wilk test for normality. *P*-value ≤ 0.05 was considered statistically significant.

## Results

### Effect of Melatonin on Runx2 Expression and NF-κB/CREB Axis in VIC Osteogenesis

**Figure 1:**
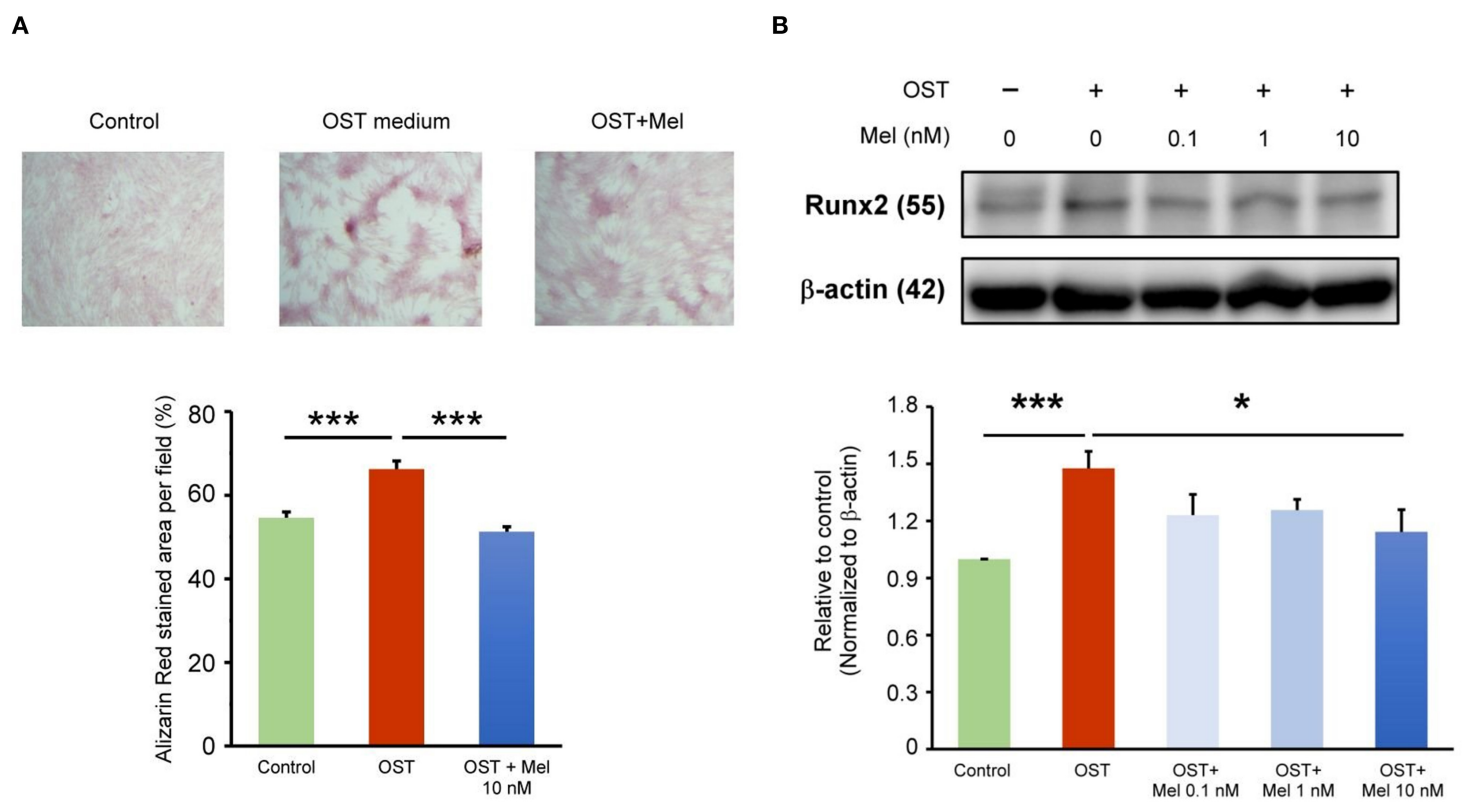
Effect of melatonin on VIC calcification. **(A)** The representative Alizarin red S staining of VICs with or without melatonin (10 nmol/L). We quantified the positive stain area (red color) per field (three fields per treatment) in the VICs (*n* = 5). The right panel presents the summary data of the percentage of calcification area (*n* = 5). ***P < 0.005. **(B)** Western blotting for melatonin on Runx2 protein expression in OST medium treated VICs for five days (*n* = 5). β-actin was used as an internal control. Data are presented as mean ± SEM. OST, osteogenic medium; Mel, melatonin. * *P* < 0.05, *** *P* < 0.005.

As shown in Figure 1A, we analyzed the effects of the OST medium on VIC calcification and determined that it significantly increased VIC calcification, as measured using ARS staining. Melatonin at 10-nmol/L significantly reduced OST medium-induced VIC calcification. As shown in Figure 1B, the expression levels of Runx2, the calcification marker, were increased in VICs treated with the OST medium for 5 days compared with the control. However, the OST medium mediation of the upregulation of Runx2 was attenuated by melatonin at 10 nmol/L [but not at 0.1 (*p* = 0.11) or 1 nmol/L (*p* = 0.181)], indicating that melatonin can inhibit OST medium induced Runx2 upregulation and VIC osteogenesis.

**Figure 2:**
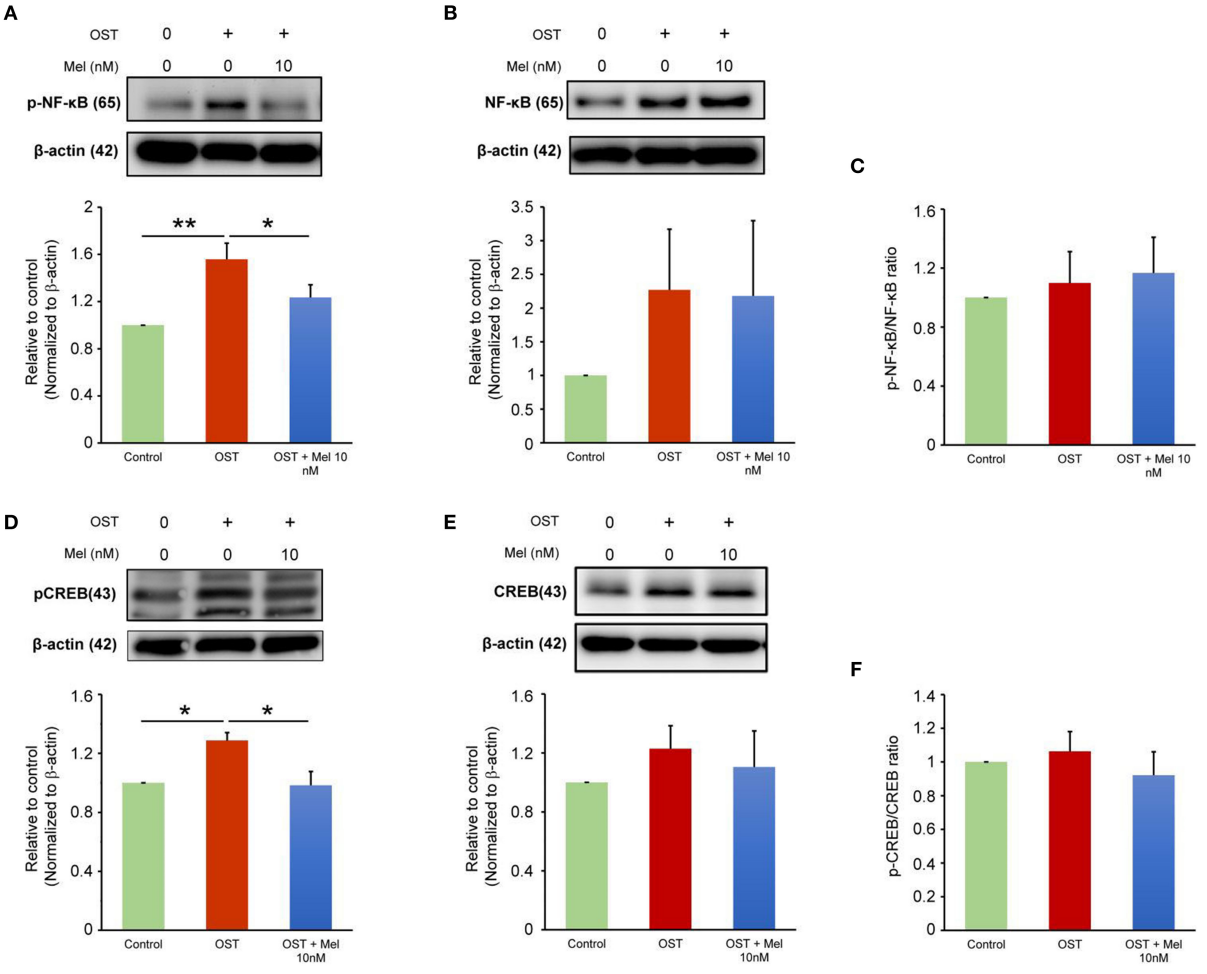
Effect of melatonin on NF-κB and CREB signaling. **(A–C)** Representative Western blots and summary data for the effect of melatonin on pNF-κB and NF-κB protein expression in OST medium-treated VICs with or without melatonin (10 nmol/L) administration for 5 days (*n* = 7). β-actin was used as an internal control. **(D–F)** Representative Western blots and summary data for the effect of melatonin on the levels of pCREB and CREB in OST medium treated VICs with or without melatonin (10 nmol/L) for 5 days (*n* = 6). β-actin was used as an internal control. Data are presented as mean ± SEM. OST, osteogenic medium; Mel, melatonin. *P < 0.05, **P < 0.01.

The previous study has shown that melatonin inhibits NF-κB transcriptional activity once translocating to the nucleus (17). The OST medium incubation increased the expression of pNF-κB and pCREB but did not increase the ratio of pNF-κB to NF-κB or pCREB to CREB as compared to the control (Figure 2). The OST medium-mediated activation of NF-κB/CREB signaling was attenuated in the VICs treated with melatonin (10 nmol/L), indicating that melatonin inhibited OST medium-induced activation of the NF-κB/CREB pathway.

### Effects of Melatonin and NF-κB Inhibitor on VIC Calcification

**Figure 3:**
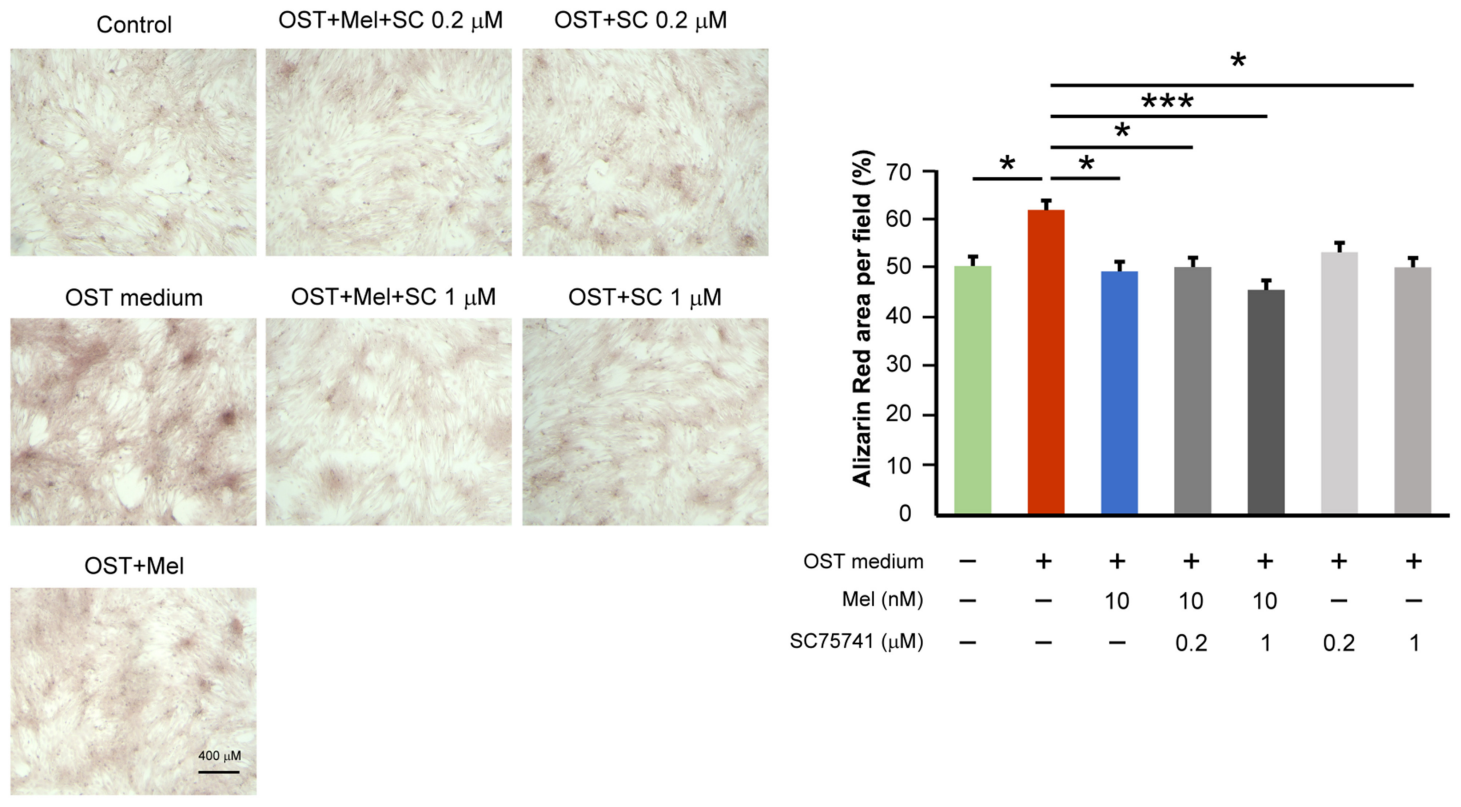
Effect of melatonin and SC75741 on VIC calcification. The left panel depicts representative Alizarin red S staining of VICs with or without melatonin (10 nmol/L) or with or without SC75741 (0.2 and 1 μmol/L). We quantified the positive stain area (red color) per field (three fields per treatment) in the VICs (*n* = 5). The right panel presents the summary data of the percentage of calcification area (*n* = 5). OST vs. OST + Mel 10 nM +SC75741 1 μM: *P* = 0.001. OST vs. OST + Mel 10 nM: *P* = 0.015. OST vs. OST + SC75741 1 μM: *P* = 0.026. OST vs. OST + Mel 10 nM + SC75741 0.2 μM: *P* = 0.027. OST vs. negative control: *P* = 0.032. OST, osteogenic medium; Mel, melatonin; SC, SC75741; *P < 0.05, ***P < 0.005.

To assess whether melatonin inhibits VIC calcification in the OST medium through its effect on downregulated NF-κB activity, we treated VICs in the OST medium with SC75741 (an inhibitor of NF-κB). SC75741 dose-dependently significantly reduced VIC calcification in the OST medium at 0.2 and 1 μM (Figure 3, Supplementary Figure 2 for all images of acquired fields). Melatonin further reduced VIC calcification in the combination treatment of SC75741 (1 μM) but not SC75741 (0.2 μM). These findings suggest that melatonin may reduce OST medium-induced VIC calcification through NF-κB activity inhibition.

### Melatonin/MT1 Axis Inhibits OST Medium-Mediated VIC Calcification

**Figure 4:**
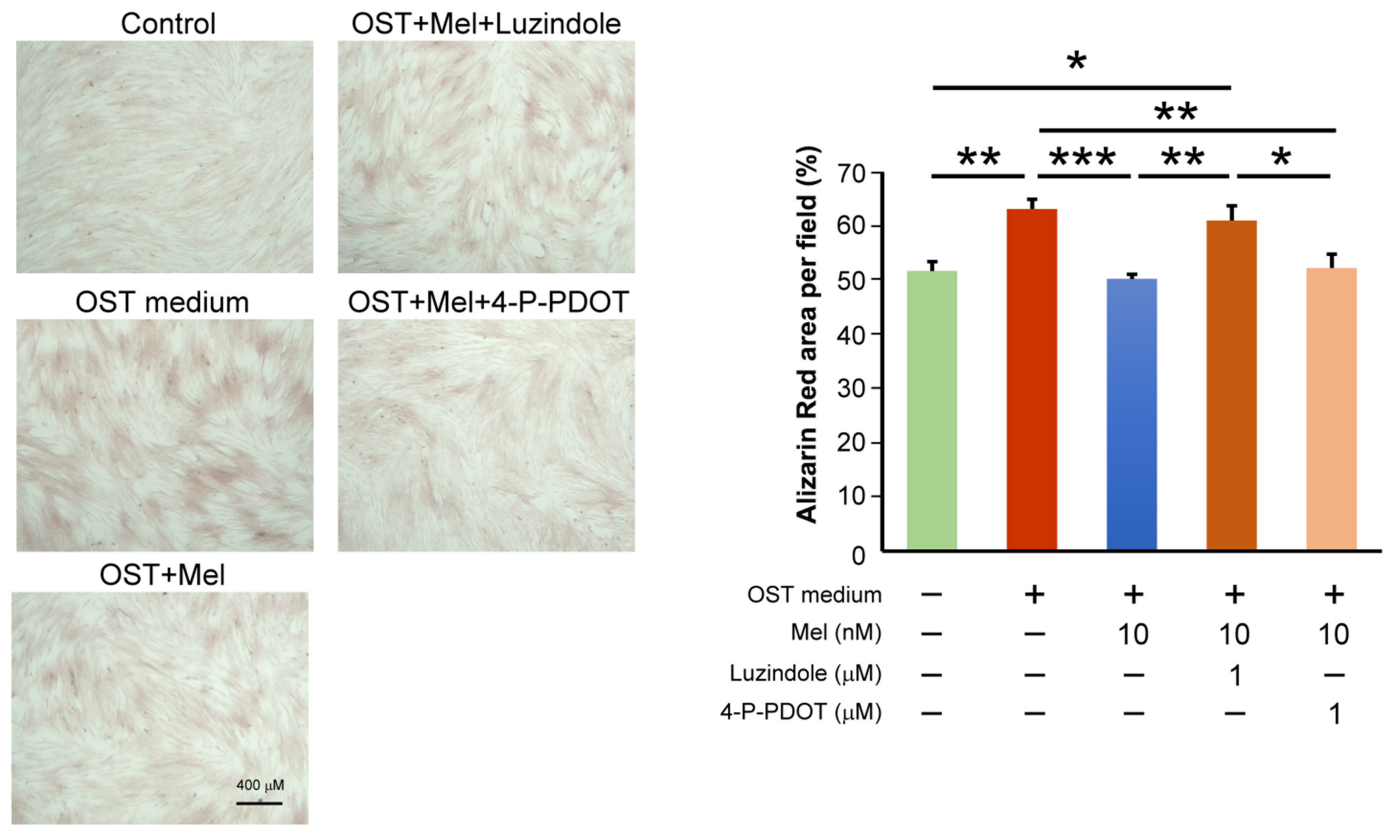
The left panel depicts representative Alizarin red S staining of VICs with or without melatonin (10 μmol/L); with or without Luzindole (1μmol/L); or with or without 4-P-PDOT (1 μmol/L). The positively stained area (red color) was quantified using ImageJ. We quantified the stain-positive area ratio per field (three fields per sample) in the VICs (n = 8). The right panel presents the average data of the percentage of calcification area (n = 8). OST vs. OST + Mel 10 nM: *P* = 0.001. OST vs. negative control: *P* = 0.005. OST vs. OST + Mel 10nM + 4-P-PDOT 1 μM: *P* = 0.008. OST + Mel 10 nM vs. OST + Mel 10 nM + Luzindole 1 μM: *P* = 0.008. OST + Mel 10 nM + Luzindole 1 μM vs. negative control: *P* = 0.028. OST + Mel 10 nM + Luzindole 1 μM vs. OST + Mel 10 nM + 4-P-PDOT 1 μM: *P* = 0.043. OST, osteogenic medium; Mel, melatonin. * *P* < 0.05, ** *P* < 0.01, *** *P* < 0.005.

**Figure 5:**
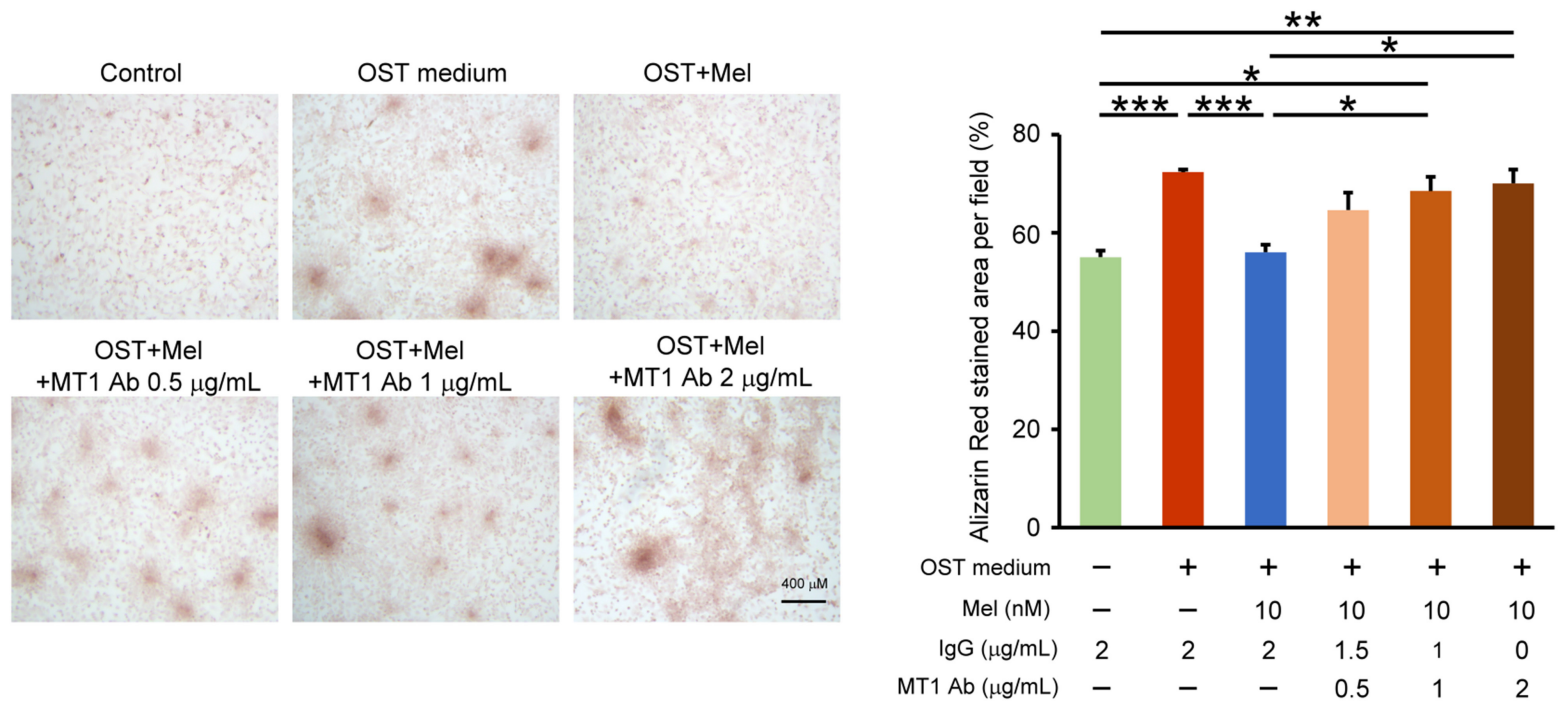
Effect of MT1 neutralized antibody on melatonin-attenuated VIC calcification. The left panel depicts representative Alizarin red S staining of VICs with or without melatonin (10 nmol/L) or with or without MT1 neutralized antibody (MT1 Ab, 0.5, 1 and 2 μg/mL) or control IgG (0.5, 1 and 2 μg/mL). We quantified the positive stain area (red color) per field (three fields per treatment) in the VICs (*n* = 4). The right panel presents the summary data of the percentage of calcification area (*n* = 4). Control (Con) vs OST: *P* = 0.002; OST vs. OST + Mel 10nM: *P* = 0.003; OST + Mel 10nM vs. OST + Mel 10nM + MT1 Ab 1 μg/mL: *P* = 0.027; Con vs. OST + Mel 10nM + MT1 Ab 1 μg/mL: 0.016; OST + Mel 10nM vs. OST + Mel 10nM + MT1 Ab 2 μg/mL: *P* = 0.012; Con vs. OST + Mel 10nM + MT1 Ab 2 μg/mL. *P* = 0.007. OST, osteogenic medium; Mel, melatonin; MT1, Melatonin receptor 1. *P < 0.05, **P < 0.01, ***P < 0.005.

We determined the effects of melatonin on VIC calcification in the presence of Luzindole (MT1/MT2 receptor inhibitor) or 4-P-PDOT (MT2 receptor inhibitor) and found that the anticalcification effects of melatonin were attenuated in the combined administration of Luzindole but not 4-P-PDOT (MT2 receptor inhibitor, Figure 4, Supplementary Figure 3 for all images of acquired fields). Moreover, the treatment of MT1 neutralized antibody also attenuated the melatonin inhibition effect on OST medium-induced VIC calcification (Figure 5, Supplementary Figure 4 for all images of acquired fields).

### Activated NF-κB Binding With the Runx2 Promoter

**Figure 6:**
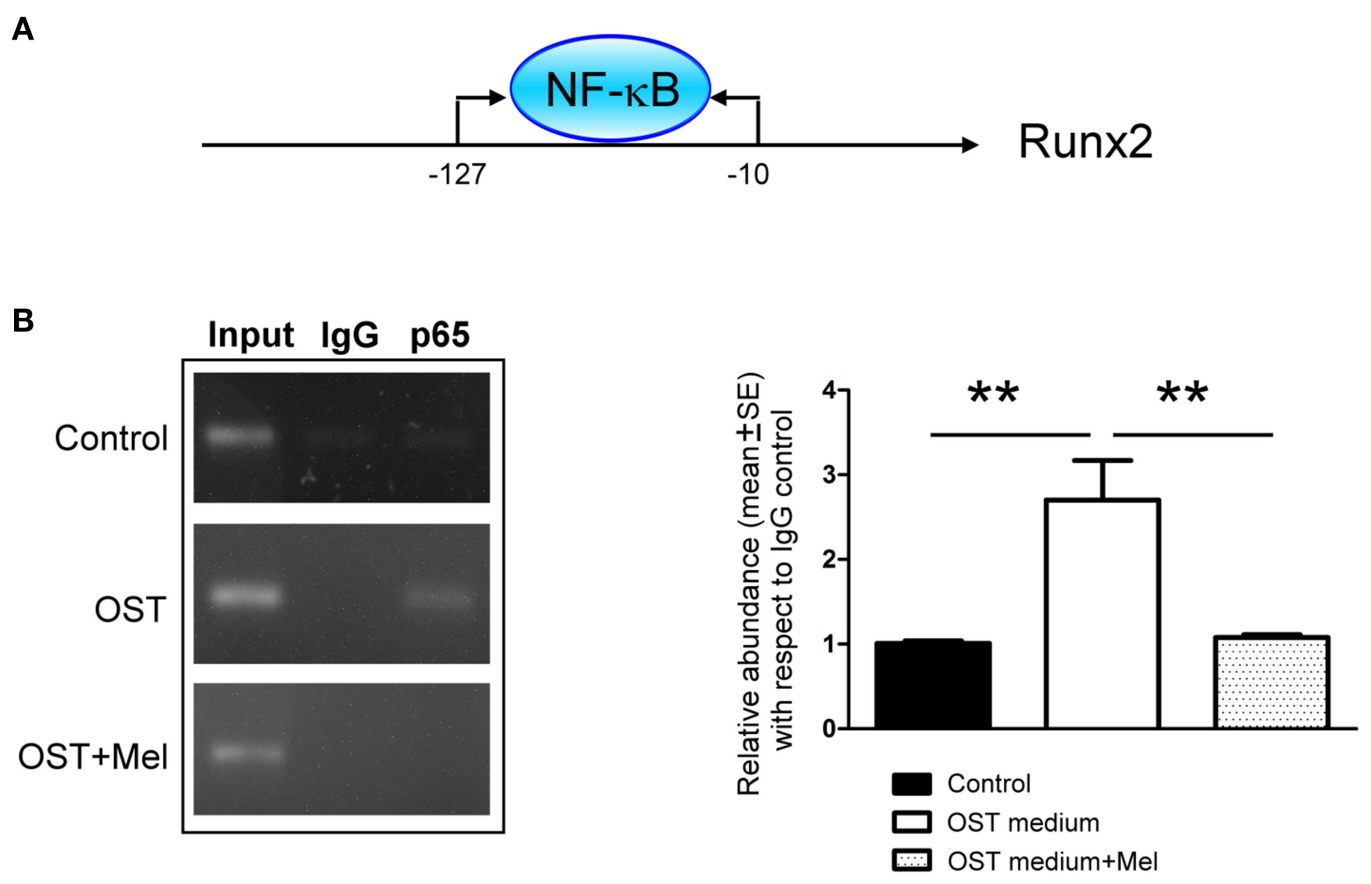
NF-κB-binding activity on the promoter region of Runx2 was determined by a ChIP assay **(A)** Schematic of the PCR amplification site of Runx2 promoter regions **(B)** Representative PCR products and summary data after ChIP assay (*n* = 4). We retained 1% of total DNA as the input control DNA. Data are presented as mean ± SEM. OST, osteogenic medium; Mel, melatonin. ** *P* < 0.01.

The ChIP assay showed that the OST media increased NF-κB p65 subunit binding to the Runx2 promoter. Moreover, its effect could be attenuated by pre-treatment with melatonin (Figure 6).

## Discussion

Our study confirmed that melatonin could diminish OST medium-induced VIC calcification and block the NF-κB/CREB/Runx2 pathway. Additionally, this study demonstrated that activation of the melatonin/MT1 axis reduced the phosphorylation of NF-κB and CREB, which are the critical transcription factors of the Runx2 promoter. Accordingly, melatonin may downregulate Runx2 through the inhibition of the NF-κB/CREB complex activity in OST medium-treated VICs. The clinical trial study results validated the beneficial therapeutic effects of melatonin (10-mg tablets/day for 24 weeks) as a combined therapy for heart failure and the patients' comorbidities (18). These findings demonstrate the therapeutic potential of melatonin for CAVD.

Porcine valves are broadly used for valve interstitial cell isolation and are a well-established model for *in vitro* study (19). However, there is a lack of good animal models of CAVD (20–22). Besides, it takes at least 16 weeks to develop an aortic valve stenosis animal model, and the mortality rate of this model is high (19, 23). The calcium content of the VIC culture increased from day three and reached a plateau after day five (24). Calcification was also demonstrated in the Master's group on day five (25). In the Balachandran group, the nodule formation in VICs was identified on day three (26). We have performed the same protocols for days three to fourteen, and finally, we chose a 5-day culture condition for this study. However, it is unclear whether a longer incubation time, up to 14 days, may change the findings in our models. Theoretically, it is supposed to have more calcification in VICs due to more apoptosis and a stronger impact of NF-κB/Runx2 signaling during longer cell cultures.

TNF-α enhanced inorganic phosphate-mediated aortic smooth muscle cell calcification by inducing the nuclear translocation of NF-κB (27). NF-κB inhibitor-SC75741 reduced the level of Runx2 in the nucleus (28). Additionally, NF-κB silencing repressed calcium deposition in the extracellular matrix of VICs incubated in calcifying media (28). Using an NF-κB binding assay, Raaz *et al*. reported that RelA (p65) binds to the predicted binding site on the Runx2 promoter (7). Using the ChIP assay, we confirmed that NF-κB (p65) could bind to the promoter region of the Runx2 gene. These findings indicate that melatonin could inhibit Runx2 expression by targeting NF-κB signaling.

Numerous studies have revealed that melatonin inhibits NF-κB activity via various mechanisms (29–34). For instance, melatonin inhibits NF-κB activation in prostate cancer cells through the MT1 receptor (35). It could directly or indirectly inhibit NF-κB biofunctions by inhibiting the ubiquitin-proteasome system (27). Melatonin can also inhibit the NF-κB translocation to the nucleus (34). It enhanced the fisetin-induced inhibition of the translocation of NF-κB p50/p65 and p300 (36). Cyclic AMP response element-binding protein-binding protein (CBP), and p300 is the transcription coactivator of p65 (37, 38). Koopmans et al. reported that β-catenin interacts with either CBP or P300, which are required for transcriptional output by p65 (39). Melatonin treatment significantly repressed the phosphorylation of p38 MAPK, JNK, and IκB-α; inhibited the binding activities of c/EBPβ and NF-κB to promoters; and repressed p300-mediated NF-κB acetylation (40). Elevated NF-κB activity increases the calcification of aortic valves (19). Caffeic acid phenethyl ester could ameliorate calcification in human aortic VICs by inhibiting the activation of the AKT/NF-κB/NLRP3 inflammasome pathway (41). Besides, targeting the NF-κB/AKT/ERK pathway using andrographolide can also inhibit the calcification of human aortic VIC (42). Recently, melatonin has been proved to ameliorate aortic valve calcification through the circular RNA CircRIC3/miR-204-5p/DPP4 signaling in VICs (43). Our novel finding demonstrated that melatonin could decrease OST-mediated VIC calcification by targeting MT1/NF-κB/Runx2 signaling.

**Figure 7:**
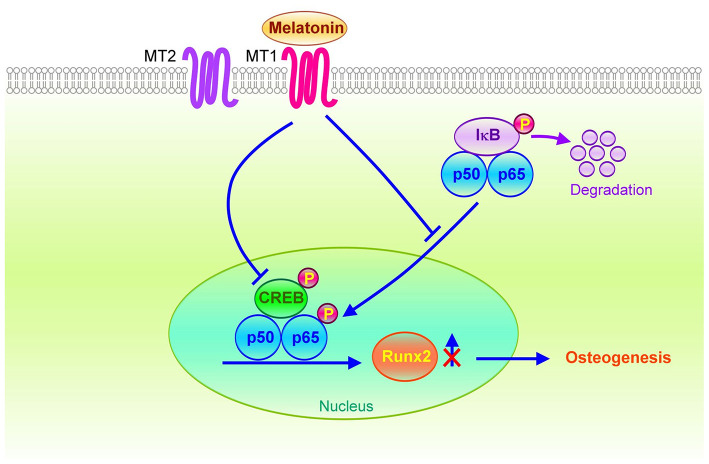
Schematic of the potential mechanisms for the therapeutic effects of melatonin on osteogenesis in VICs. The melatonin/MT1 axis may downregulate Runx2 expression through inactivation of NF-κB/CREB signaling in OST medium-treated VICs. Conversely, activation of the NF-κB/CREB complex may bind to the Runx2 promoter to increase Runx2 gene transcription through VIC osteogenesis.

A study determined that bone morphogenetic protein 9 (BMP9) activated CREB signaling and induced osteogenic differentiation of mesenchymal stem cells (44). Decreasing CREB signaling could attenuate BMP9-induced osteogenesis. In this study, we identified that the inhibition of MT1/MT2 activities by Luzindole could diminish the effect of melatonin on reducing VIC calcification and inactivation of NF-κB/CREB/Runx2 signaling. However, the MT2-specific antagonist-4P-PDOT did not significantly inhibit the activity of melatonin. As presented in Figure 7, OST media administration may induce NF-κB and CREB synergistically binding to the promoter region of Runx2, causing the upregulation of the downstream Runx2 gene and promoting osteogenesis. However, activating the melatonin/MT1 axis could diminish OST media-induced upregulation of NF-κB/CREB signaling.

## Conclusions

Activation of the melatonin/MT1 axis significantly reduced VIC calcification through the targeting of the NF-κB/CREB/Runx2 pathway. The initiation of melatonin signaling may be a potential therapeutic strategy for CAVD progression.

## Data Availability Statement

The original contributions presented in the study are included in the article/Supplementary Materials, further inquiries can be directed to the corresponding author.

## Ethics Statement

The animal study was reviewed and approved by Taipei Medical University's Institutional Animal Care and Use Committee (LAC-2020-0332).

## Author Contributions

S-JL designed the whole study, performed the experiments, and analyzed the data. Y-JC coordinated the study. W-LC, Y-HK, C-CC, and Y-JC wrote and modified the manuscript. All authors have discussed the manuscript text and figures. All authors have approved the final manuscript.

## Funding

This work was funded by grants from Wan Fang Hospital (107TMU-WFH-01-1 and 108TMU-WFH-01-3), Taipei Medical University (TMU109-AE1-B09), and the Ministry of Science and Technology (Taiwan, Grant No. MOST108-2314-B-038-120).

## Conflict of Interest

The authors declare that the research was conducted in the absence of any commercial or financial relationships that could be construed as a potential conflict of interest.

## Publisher's Note

All claims expressed in this article are solely those of the authors and do not necessarily represent those of their affiliated organizations, or those of the publisher, the editors and the reviewers. Any product that may be evaluated in this article, or claim that may be made by its manufacturer, is not guaranteed or endorsed by the publisher.
