# Supplementary material for: Melatonin Inhibits NF-κB/CREB/Runx2 Signaling and Alleviates Aortic Valve Calcification
Source: Front Cardiovasc Med. 2022 Jun 20;9:885293. doi: 10.3389/fcvm.2022.885293 (PMC9251177; doi:10.3389/fcvm.2022.885293)
Supplement: Supplementary file 1 [file Data_Sheet_1.PDF]

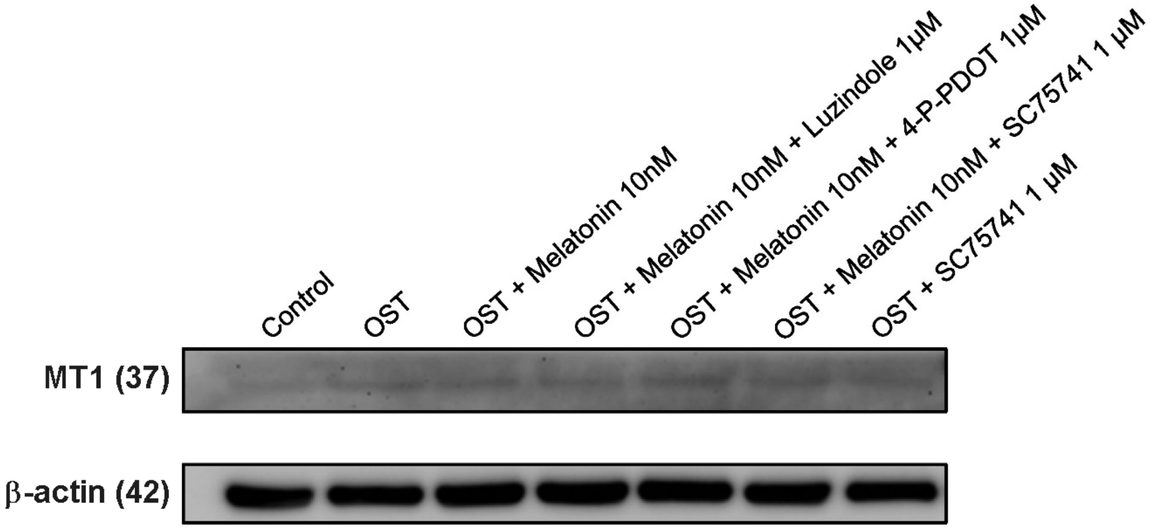

Supplementary Figure 1. Melatonin receptor 1 (Mel-1A-R; 1:5000; Cat# sc-390328, Santa Cruz) exists in porcine VICs. OST: osteogenic medium.

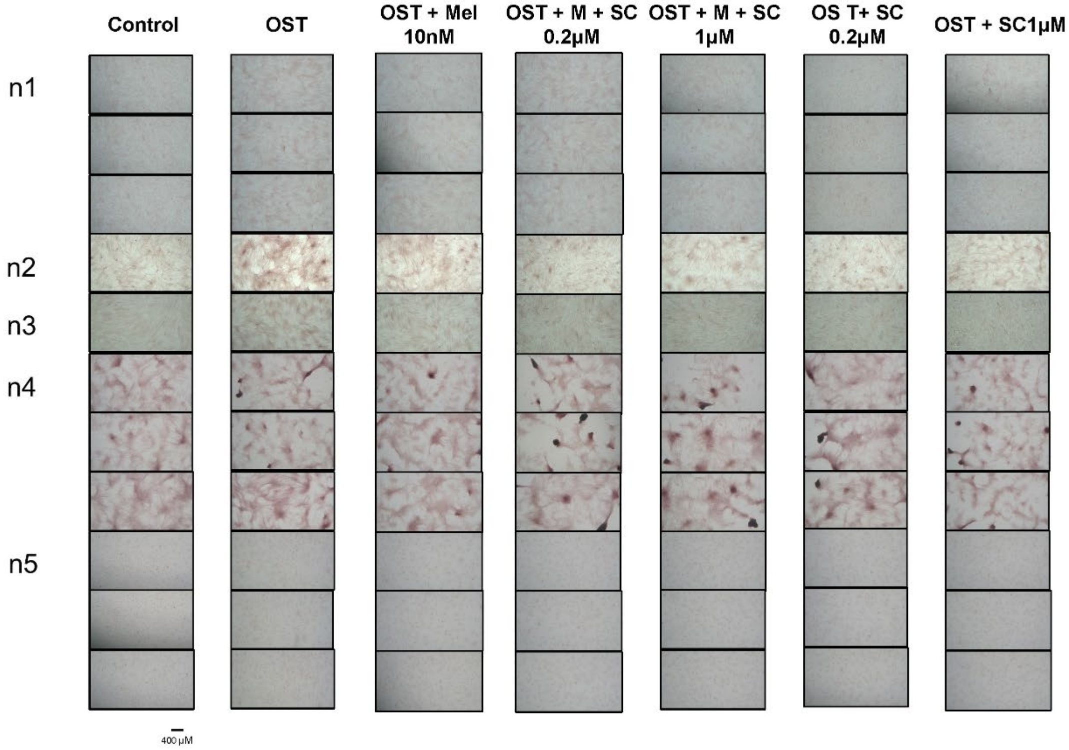

Supplementary Figure 2. All the images of acquired fields in Figure 3 (Effect of melatonin and SC75741 on VIC calcification). OST: osteogenic medium. Mel or M: melatonin. SC: SC75741, NF-κB inhibitor. The number along the side, n1-n5, indicates the label of each specimen (n = 5).

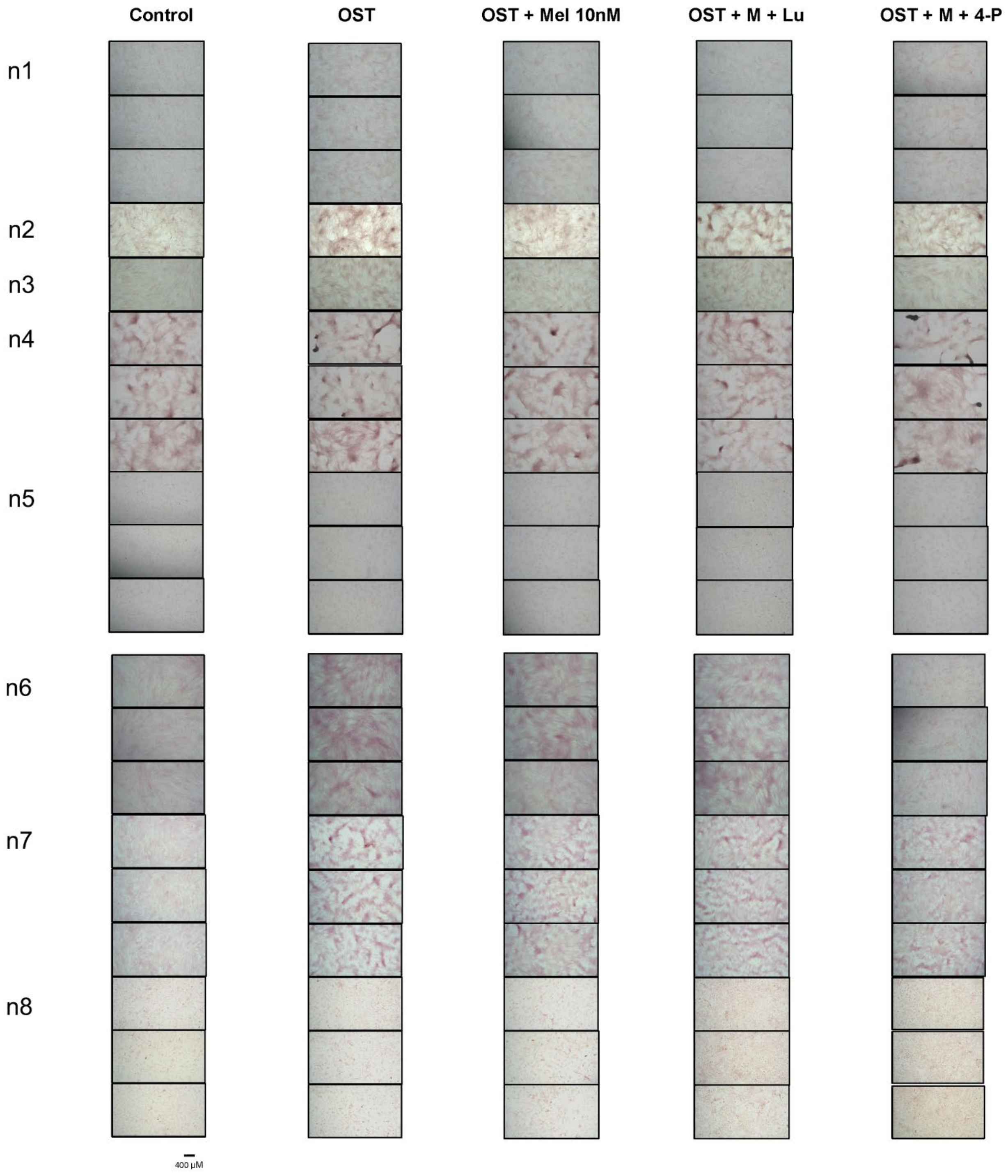

Supplementary Figure 3. All the images of acquired fields in Figure 4 (Effects of melatonin receptor antagonists on VIC calcification). The number along the side, n1-n8, indicates the label of each specimen (n = 8). OST: osteogenic medium. Mel or M: melatonin. Lu: Luzindole. 4-P: 4-P-PDOT

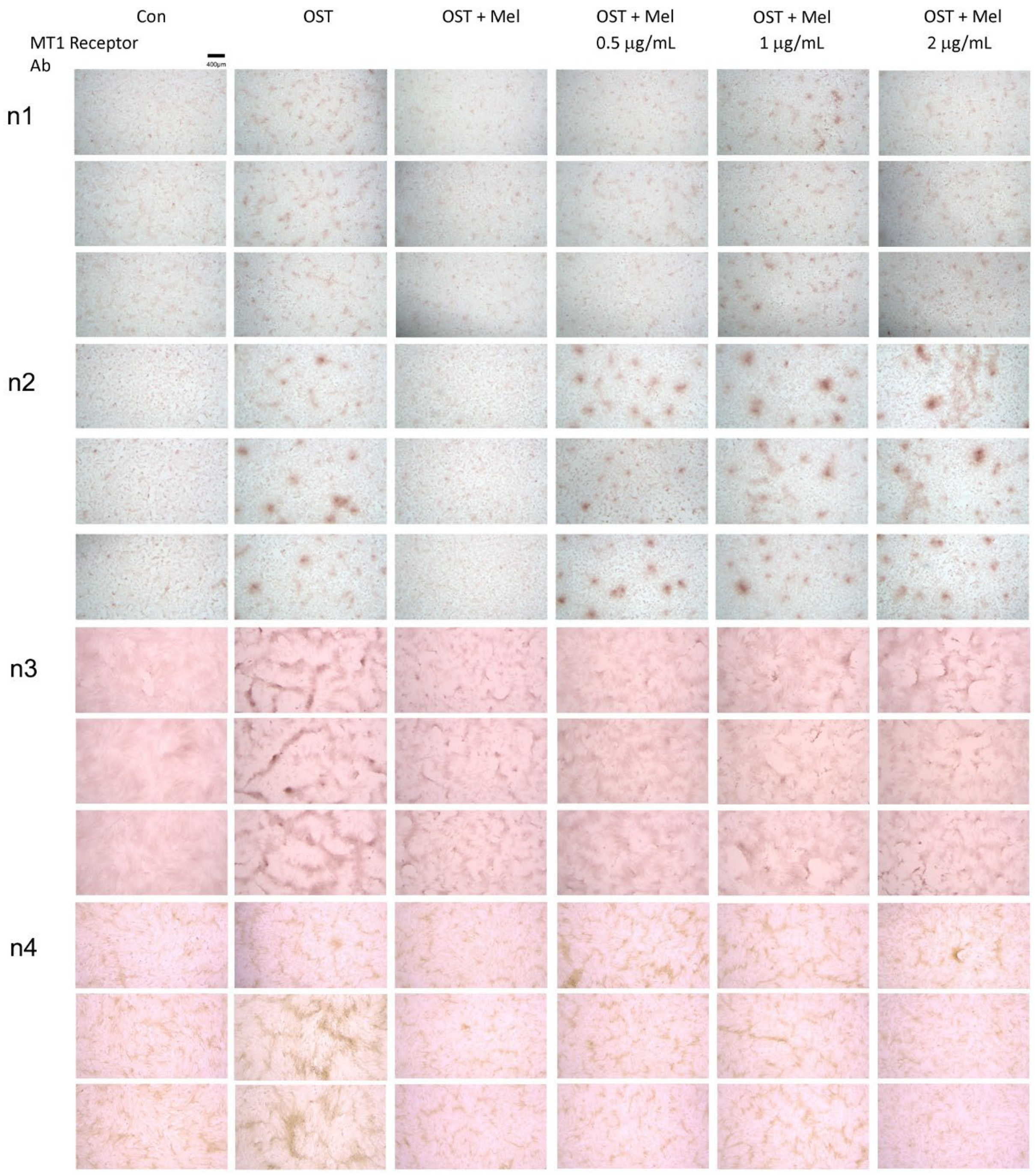

Supplementary Figure 4. All the images of acquired fields in **Figure 5** (**Effects of MT1 neutralized antibody on melatonin-attenuated VIC calcification**). The number along the side, n1-n4, indicates the label of each specimen (n = 4). OST: osteogenic medium. Mel: melatonin. MT1: Melatonin receptor 1.
